# Reviewing the complex relationship between circadian rhythms and cluster headache

**DOI:** 10.1177/03331024251365858

**Published:** 2025-08-14

**Authors:** Mark Burish, Seung Ae Kim, Caroline Ran, Seung-Hee Yoo, Mi Ji Lee, Andrea Carmine Belin

**Affiliations:** 1 Department of Neurosurgery, UTHealth Houston, Houston, TX, USA; 2 Department of Neurology, Seoul National University Hospital, Seoul National University College of Medicine, Seoul, Republic of Korea; 3 Graduate School of Translational Medicine, Seoul National University College of Medicine, Seoul, Republic of Korea; 4 Centre for Cluster Headache, Department of Neuroscience, Karolinska Institutet, Stockholm, Sweden; 5 Department of Biochemistry and Molecular Biology, UTHealth Houston, Houston, TX, USA

**Keywords:** chronobiology, hypothalamus, pain, sleep

## Abstract

Cluster headache attacks display uniquely rhythmic patterns in their manifestations. Multiple international studies have shown circadian and even circannual timing of attacks, although we do not yet fully understand the effects of culture, sleep, chronotype, seasonal changes, temperature or inter-individual changes over time. Multiple cluster headache treatments alter the core circadian oscillator, although they affect the oscillator differently and are not well understood. Multiple small genetic studies have shown core circadian gene variants to be cluster headache susceptibility genes, whereas larger genetic studies have not shown core circadian gene variants but have also not documented the presence or absence of circadian rhythmicity. In this narrative review, we describe the multi-level circadian features of cluster headache and propose future circadian directions, including a clinical definition of circadian attacks, a potential animal model of circadian headache and study design changes to incorporate circadian features into larger genetic studies.

## Introduction

We start with a clinical vignette: “A patient presents with headache attacks that occur twice a day at almost exactly 4:00 pm and 2:00 am every day. What is the most likely diagnosis?” This question has been posed to audiences of headache specialists throughout the years by some of the authors of the current review. Despite providing only two full pieces of information (frequency and timing), minimal information on location (“headache”) or duration (twice in one day), and no information of severity, associated features or demographics, headache specialists recognize this as cluster headache (CH). While several other headache disorders can occur twice a day and hypnic headache follows a 24-hour pattern but only during sleep, we posit that CH stands unique among headache disorders in its relationship to the circadian rhythm.

The circadian clock, or our internal 24-hour timer, regulates innumerable physiologic processes from the sleep–wake cycle to daily blood pressure fluctuations. Disruption of the circadian clock can have noticeable health impacts: shift workers, for example, have an increased risk of diabetes and myocardial infarction (1). Shift working, of note, has also been reported to occur more frequently in CH cohorts than what is usually seen in the general population (2,3). By contrast, understanding the circadian clock can lead to great health benefits. Specific time-of-day administration of treatments (the “clocking the drugs” form of chronotherapy) (4–6) has been shown to improve outcomes in patients with hypertension, rheumatoid arthritis, metabolism and many other diseases (7). There are data suggesting that verapamil, the first-line prophylactic treatment for CH, could affect the circadian phenotype of CH by slightly shifting the time of attacks forward (8). Meanwhile altering the circadian clock at the molecular level (the “drugging the clock” form of chronotherapy) may play an important role not only in circadian and sleep disorders, but also in cancer and other areas (4). We will discuss the molecular circadian clock below because these genes are highly relevant to CH pathophysiology, genetic research, and potential future treatments.

The circadian system in humans, at the most fundamental level, is a single-celled clock. Almost all human cells contain a transcriptional-translational feedback loop of core circadian proteins and genes that activate and inhibit each other in a process that takes approximately 24 hour (9). This single-celled clock receives inputs, called zeitgebers (for environmental cues like light) or chronotherapeutics (for drugs), which resynchronize or alter the clock. This single-celled clock then provides outputs, called clock-controlled genes, that ultimately regulate the sleep–wake cycle, blood pressure and other physiological processes.

As there are trillions of single-celled clocks in a person, they must be synchronized. The master pacemaker for the body is located in the suprachiasmatic nucleus of the hypothalamus. The suprachiasmatic nucleus receives light information from the retina in the eye via the retinohypothalamic tract and orchestrates the clocks of other tissues. Signalling for the circadian system is the same as that for many other biological processes: hormonal and neuronal signals, with the primary hormones being corticosteroids (daytime signal) and melatonin (nighttime signal) via the suprachiasmatic nucleus’ connections with the hypothalamic–pituitary–adrenal axis and pineal gland. The circadian signalling of specific proteins and/or organs leads to its innumerable physiologic effects.

In this narrative review, we provide an overview of the emerging evidence of circadian features in CH, their potential underlying pathophysiology and the chronotherapeutic properties of many CH medications. The ultimate goal is that, in understanding the circadian disruptions in CH, we can ultimately improve CH outcomes.

## Circadian and seasonal rhythmicity

This section outlines the chronobiological patterns of CH, focusing on circadian and seasonal rhythmicity, their variability and potential clinical implications. Detailed findings from individual studies are presented in Table 1.

### Circadian rhythmicity

**Figure 1.**
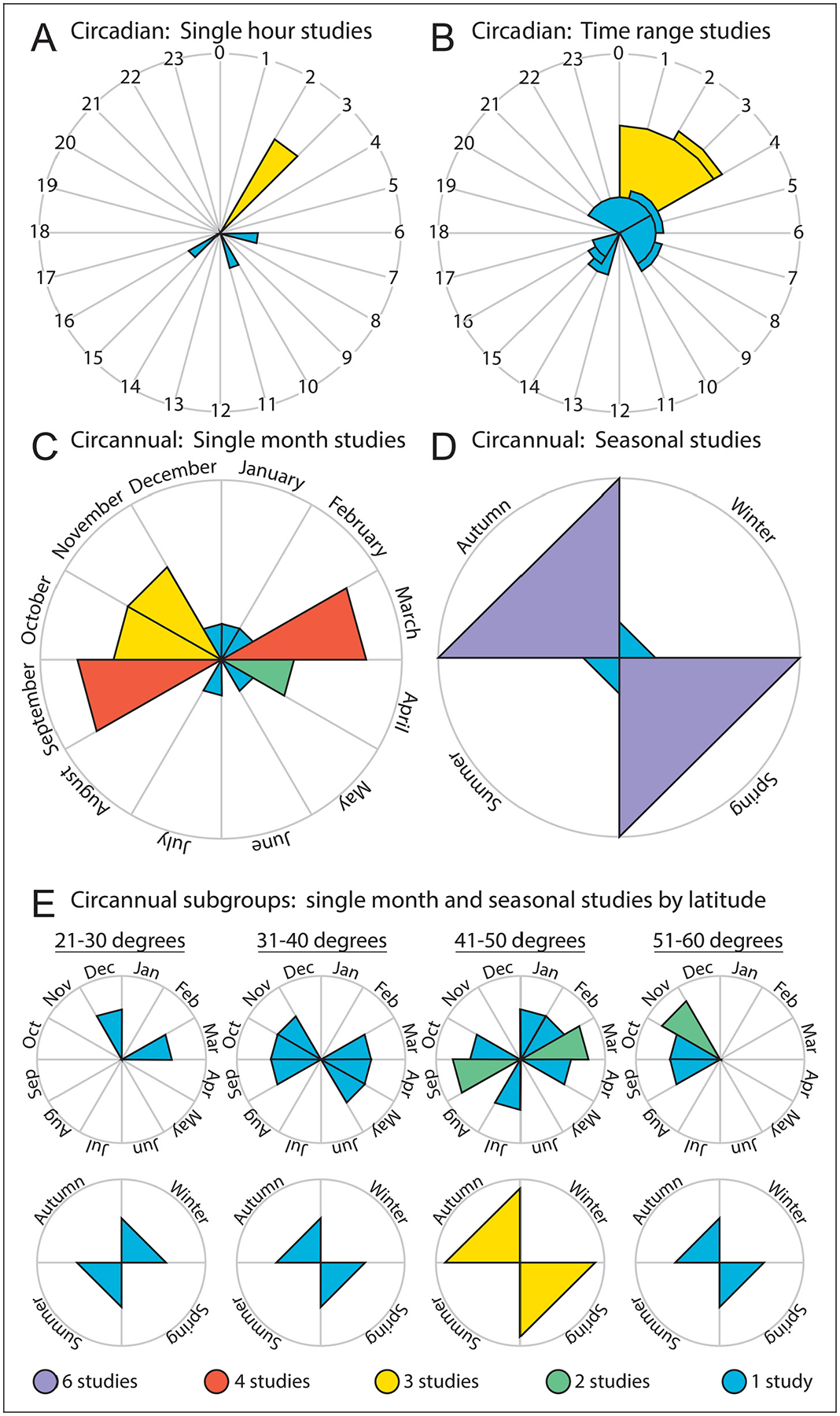
Summary of studies on the daily (circadian) and yearly (circannual) timing of cluster headache. Colours reflect the number of independent studies with the same result, with colour legend at bottom. (A) Studies reporting hour-by-hour circadian results. (B) Studies reporting hourly circadian ranges. (C) Studies reporting month-by-month circannual results. (D) Studies reporting seasonal circadian results. (E) Seasonal subgroup results for studies at different latitudes. Latitudes indicate the average latitude of a country’s northern-most and southern-most major city as reported by the United Nations. For details, see Table 1

The presence of circadian rhythmicity in CH has been reported in approximately 70% of patients with CH from a meta-analysis of 16 studies involving 4953 individuals (10). Between individual studies, however, significant variability was noted in the prevalence of circadian rhythmicity, ranging from 49% to 87% across studies (Table 1) (2,3,10–35). In a study conducted by one of the authors of the current review, a discrepancy according to the record type was identified in the prevalence of circadian rhythmicity within the same patient group: 49.1% during the currently active bout vs. 70.9% over the lifetime bout (10,31). This finding explains that the prevalence of circadian rhythmicity, retrospectively collected in most studies based on patients’ recall regarding the overall lifetime experience, might not accurately reflect the actual cross-sectional prevalence of circadian rhythmicity, and the presence of circadian rhythmicity may have changed over time. The between-study variability may also be attributed to a lack of consensus definition of circadian rhythmicity. Taken together, a consensus on the definition of circadian rhythmicity and methodology of data collection is needed to ensure the accuracy of research. As shown in a previous meta-analysis of three retrospective studies involving 8856 patients, CH attacks can occur anytime of the day, with the highest prevalence between 2:00 am and 3:00 am (508/8856) (10). Across retrospective cohorts from Denmark, Sweden, the Netherlands and the USA, the most common peak time for CH attacks was distributed between 8:00 pm and 4:00 am (Figure 1A), with 2:00 am being the most frequent peak time (10). Peak time distribution outside this window was reported in cohorts from Norway (4:00 am to 10:00 am), Italy (1:00 pm to 3:00 pm), India (2:00 pm to 5:00 pm and 12:00 am to 4:00 am), China (7:00 am to 10:00 am and 2:00 pm to 4:00 pm) and South Korea (10:00 am, 3:00 pm and 2:00 am) (Figure 1B) (22,23,28,32,35).

Differences in circadian peak timing may be influenced by cultural routines (23,28,31,32,35), chronotype and sleep (8), and biological factors (14,17). In Italy, a peak attack timing in the early afternoon may reflect the influence of cultural practices like the midday siesta (23). In Asian populations, earlier wake times and more structured daytime schedules may contribute to more frequent diurnal or bimodal patterns (28,31,32,35). A Danish study found that the peak timing of nighttime CH attacks varied with chronotype, occurring earliest in morning types (0:50 am), slightly later in intermediate types (1:02 am) and latest in evening types (2:11 am) (8). In addition, an association was observed between poor sleep quality (Pittsburgh Sleep Quality Index > 5) and a tendency for attacks to cluster during the night, particularly around 9:46 pm, 2:16 am and 6:03 am (8). This finding suggests that poor sleep may predispose individuals to nocturnal attacks or, conversely, that frequent nocturnal attacks may lead to poor sleep quality. Among biological factors, sex-related circadian rhythmicity differences have also been explored, although findings remain inconsistent. One study found that men tend to experience attacks slightly earlier than women (14), whereas another suggested that women show stronger circadian rhythmicity and more nocturnal attacks (17). By contrast, other studies have found no significant sex-related differences (18,34).

### Circannual rhythmicity

Circannual or “seasonal” rhythmicity, a tendency for CH bouts to occur during specific times of the year, is another interesting chronobiological characteristic of CH. Across studies, 37–73% of patients reported a seasonal predilection of bouts (2,11,13,14,17,25,28,30–34). Month-level analyses across studies identified March, April, September, October and November as the most frequent months for CH onset (Figure 1C), accounting for 53.8% (1882/3495) of participants in the meta-analysis (10). This corresponds to peaks in spring and autumn (Figure 1D), with a review of 3709 patients reporting autumn (31%) as the most common peak season (2,10,11,13,17,20,25,26). Summer months, especially June, were consistently associated with lower attack frequency.

Notably, countries exhibiting these seasonal patterns, such as Denmark, Sweden, Norway, Italy, the northeastern United States (Pennsylvania) and northern China (Beijing), are predominantly located at higher latitudes, where seasonal variation in daylight is more pronounced (11,13,17,23,25,35). In these countries, seasonal rhythmicity was reported in approximately 50–56% of patients. In contrast, studies conducted in lower- or mid-latitude regions reported different patterns. In India, peaks were noted in summer (30%) and winter (16%); in the southwestern USA (California), peaks were observed in January and July (Figure 1E and F) (27,28). Proposed mechanisms for encoding seasonal rhythmicity include changes in melatonin, changes in pituitary hormones, and differences in neuronal firing in the suprachiasmatic nucleus between short days and long days (36).

Furthermore, regional variation within countries has also been observed. In the USA, seasonal peaks occurred in different months depending on region: northeastern states (e.g. Pennsylvania) showed peaks in October (26%), whereas southwestern states (e.g. California) exhibited peaks in January and July (25,27). A study in Taiwan demonstrated more pronounced rhythmicity in the northern region compared to that in the south (33). Furthermore, a strong inverse correlation between sunshine hours and CH frequency was also observed (*r* = −0.774, *p* < 0.001) (33). In addition to photoperiod, environmental factors such as temperature may also contribute to seasonal variation. Another Taiwanese study showed that cluster bouts were more likely to occur with higher mean temperatures, even after adjusting for relative humidity, atmospheric pressure, wind speed, sunshine duration and rainfall days (37). Several studies reported increased CH frequency during transitional periods, particularly from winter to spring and spring to summer, raising the possibility that temperature fluctuations act as additional triggers (11,13,37). These findings collectively suggest that both the timing and the intensity of seasonal rhythmicity may be influenced by regional environmental factors such as geographic latitude, sunlight exposure and temperature.

### Temporal changes of rhythmicity in CH

While circadian rhythmicity presumably reflects the unique involvement of the hypothalamus in CH pathophysiology, it is not consistently observed between or even within patients. Barloese et al. (38) evaluated patient-reported changes for the last three years, focusing on phenotype shifts (i.e. transitions between episodic and chronic CH) in 37% of cases. Importantly, those progressing to chronic CH exhibited increased ultradian variability, characterized by more fragmented or irregular attack timing patterns within a 24-hour cycle, while patients reverting to episodic CH showed rhythmicity patterns comparable to stable episodic cases (38).

**Figure 2.**
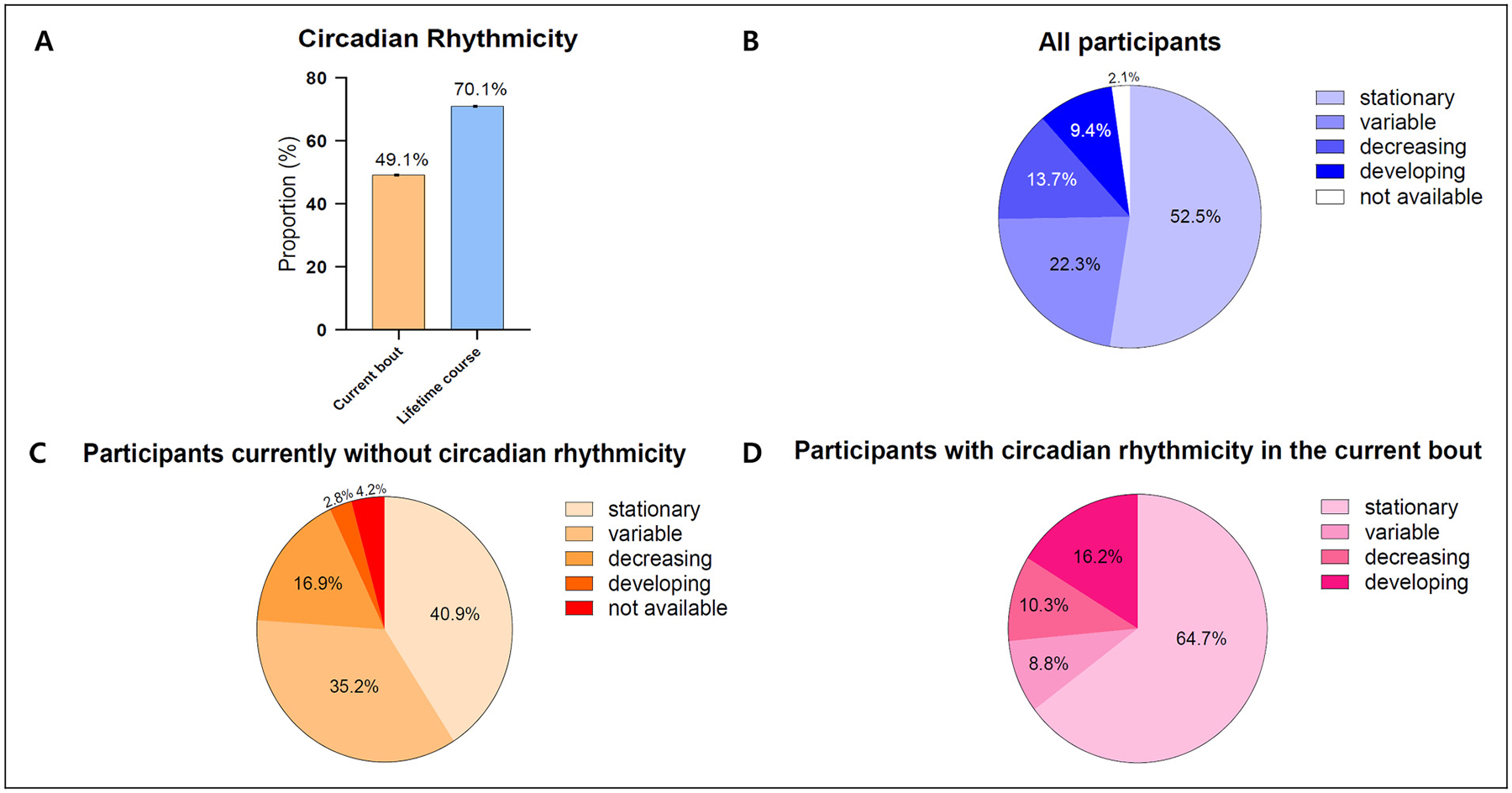
Circadian rhythmicity and bout-to-bout temporal patterns in cluster headache. (A) Proportion of participants with circadian rhythmicity in the current bout and across the lifetime course. (B) Overall temporal patterns of circadian rhythmicity in all participants with two or more bouts. (C) Temporal patterns of circadian rhythmicity in participants currently without circadian rhythmicity. (D) Temporal patterns of participants with circadian rhythmicity in the current bout (generated from the same cohort previously published by Lee et al. (31) using different visualization methods)

**Figure 3.**
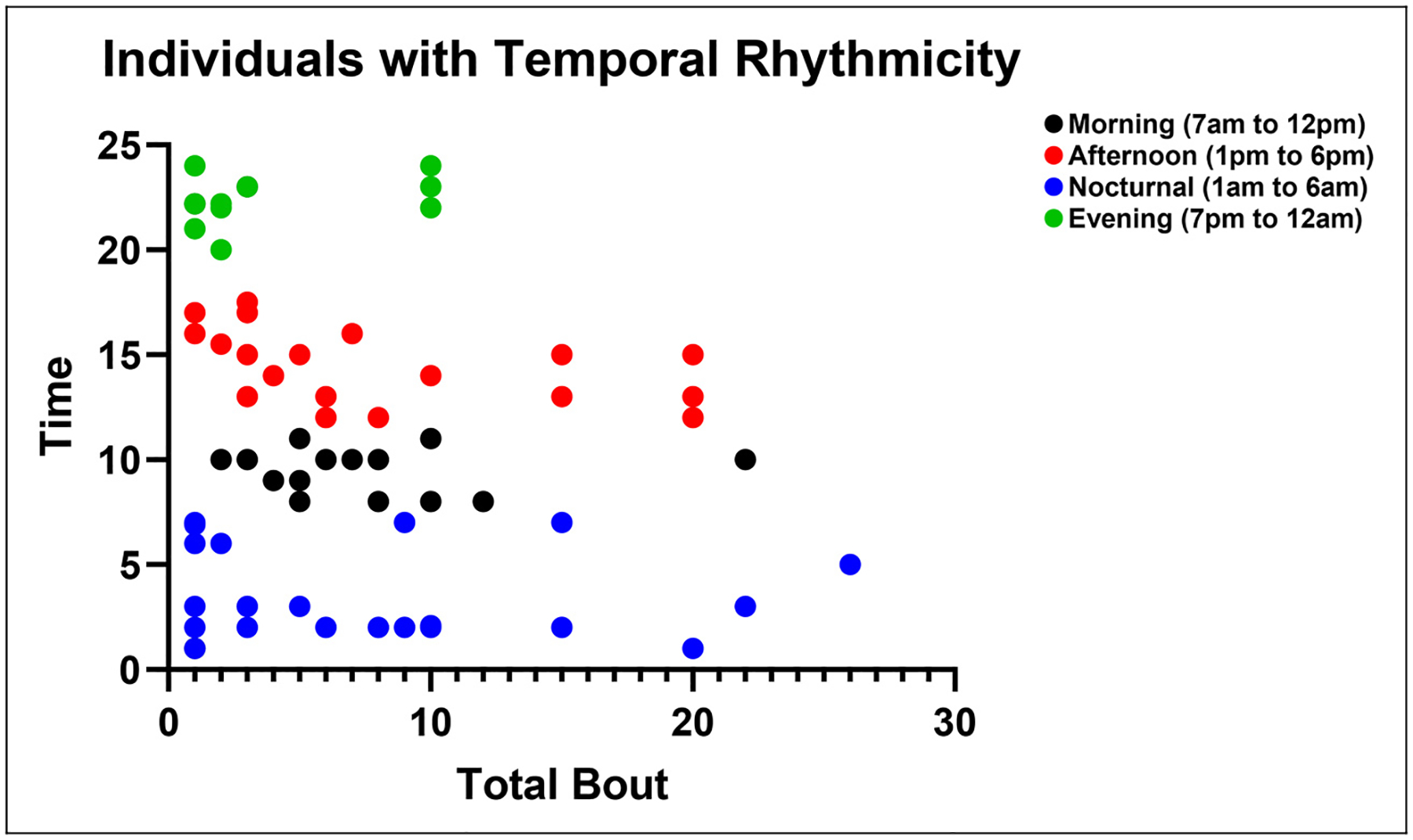
Scatterplot showing the relationship between the time of most frequent occurrence and the total number of lifetime bouts in patients with circadian rhythmicity. In the early stages of the disease, the timing of attacks was relatively evenly distributed across the 24-hour cycle. As the disease progressed, a dichotomous pattern emerged, with attack timings clustering around nocturnal and midday hours (generated from the same cohort previously published by Lee et al. (31) using different visualization methods)

**Figure 4.**
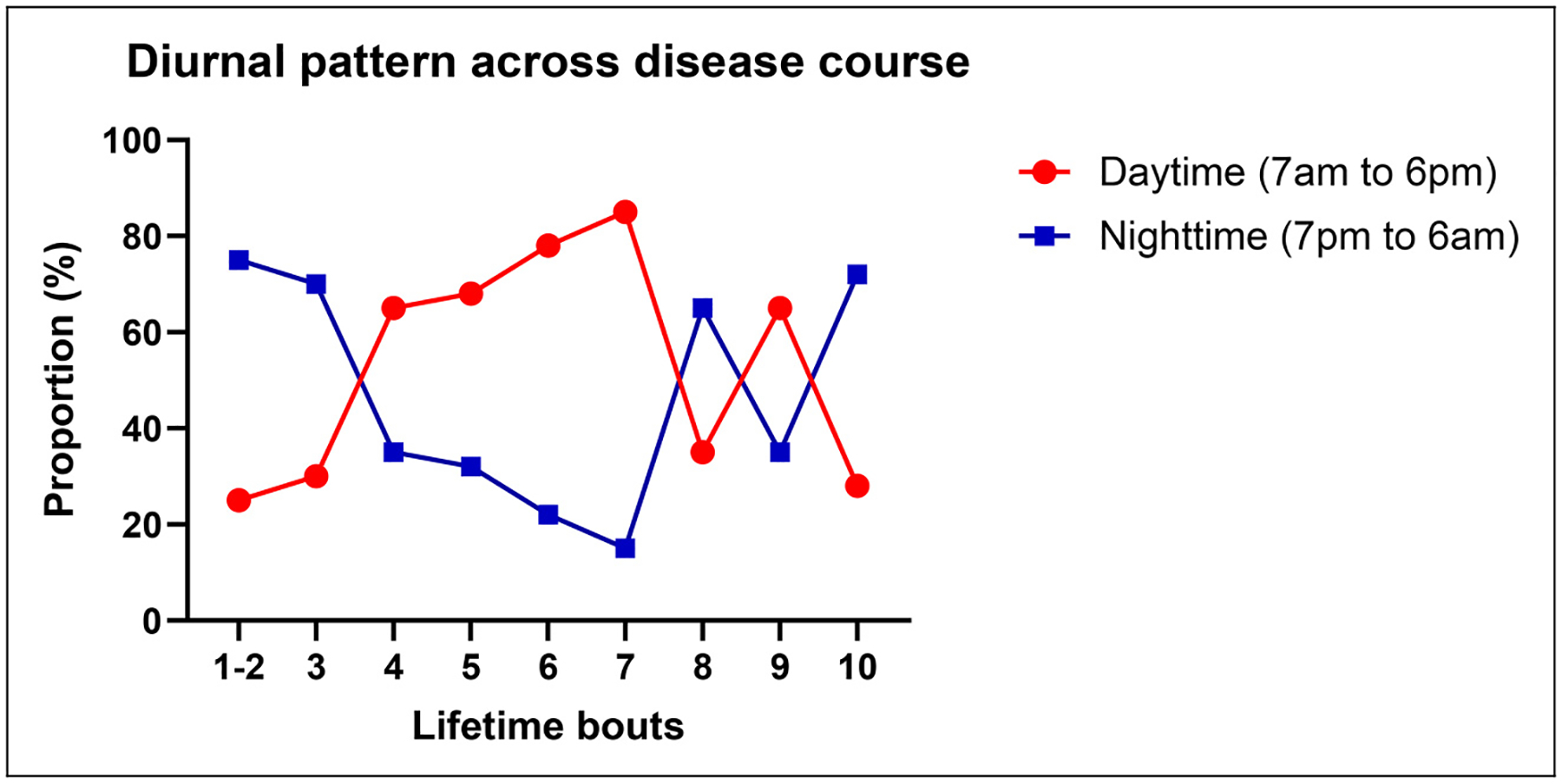
Temporal shift in diurnal attack pattern across lifetime bouts. Proportions of daytime (7:00 am to 6:00 pm) and nighttime (7:00 pm to 6:00 am) attacks are shown by bout groups. Nighttime attacks predominated early (first and second bouts), but daytime attacks increased mid-course (up to seventh bout). In later bouts (eight to tenth bouts), nighttime predilection reemerged, suggesting dynamic changes in diurnal rhythmicity over time (generated from the same cohort previously published by Lee et al. (31) using different visualization methods)

Lee et al.(31) conducted a multicenter study involving 139 patients regarding the temporal change of circadian rhythmicity in CH. In this study, 49.1% of patients showed circadian rhythmicity during the prospective observation of the currently active bout, whereas 70.9% reported circadian rhythmicity throughout their entire disease course (Figure 2A). When asked about temporal changes across previous bouts among patients with two or more lifetime bouts, 45% reported that their circadian pattern had changed over time: variability between bouts (22%), decreasing rhythmicity (14%) and increasing rhythmicity (9%) (Figure 2B), with differences according to the presence of circadian rhythmicity (Figure 2C, D) (31). When the association between timing of attacks and disease progression (i.e. the number of lifetime bouts) was examined, attack timings were evenly distributed in the earliest stage (first-onset CH: one lifetime bout). As bouts accumulated (two to 100 lifetime bouts), however, attacks progressively shifted toward either nocturnal (1:00 am to 6:00 am) or daytime (1:00 pm to 6:00 pm) periods, with a significant difference in the variance of lifetime bouts across attack timing groups (*p* = 0.037 for the homogeneity of variance) (Figure 3). Diurnal patterns showed nocturnal attacks dominated in early (one lifetime bout) and late (more than eight lifetime bouts) stages, while daytime attacks were more frequent mid-course (two to eight lifetime bouts), suggesting a non-linear and dynamic evolution of circadian expression in CH (Figure 4) (31).

Further intraindividual variability was demonstrated in a six-year prospective study by Hagedorn et al.,(39) which documented 4600 attacks in a single patient with chronic CH. Although overall circadian rhythmicity was predominant, spectral analysis revealed a temporal shift in rhythmic patterns during periods of high attack frequency, from an ultradian-dominant pattern (July 2012 to March 2014) to a circadian-dominant pattern (June 2015 to July 2016). Notably, no consistent seasonal pattern was observed because attack frequency and severity did not systematically vary across months or seasons (39).

Together, these findings suggest that circadian rhythmicity in CH is not a fixed trait but may evolve dynamically over time, influenced by disease progression, phenotype shifts and intraindividual variability.

### Clinical implications of rhythmicity in CH

The temporal features of CH, including circadian and seasonal rhythmicity, may carry important clinical implications as they are strongly associated with the hypothalamus, which is considered as a core attack generator of CH. With this regard, circadian and seasonal rhythmicity might serve as a clinical predictor of disease activity and therapeutic response. In addition, recognizing the predictable timing of attacks could help optimize both preventive and acute treatment strategies.

Recently, the concept of “chronorisk” has been introduced to quantify the probability of an attack occurring at a given time of day. Barloese et al.(8) demonstrated that patients with episodic CH typically exhibit a circadian chronorisk profile with a single early morning peak, whereas those with chronic CH display an ultradian pattern marked by multiple, less predictable peaks throughout the day. These subtype-specific chronorisk patterns may aid in distinguishing CH phenotypes and in providing personalized treatment aligned with circadian profiles (34). Circadian and seasonal rhythmicities are well-established in CH and may have clinical relevance, particularly in relation to disease activity. In a Korean registry study, Lee et al.(30) found that patients with active disease more frequently exhibited circadian and seasonal rhythmicity at baseline compared to those in long-term remission, suggesting that temporal patterns may reflect underlying disease activity. Moreover, seasonal rhythmicity was identified as a risk factor of frequent bout relapse in a five-year prospective study involving 295 patients (Lee et al., in press)(40). These observations suggest that rhythmicity may serve as a surrogate marker of disease activity.

Pre-cluster symptoms (i.e. symptoms that precede the CH bout and help predict upcoming bouts) were first identified by a Danish group, and further investigated in a Korean multicenter cohort and a Taiwanese cohort where data regarding circadian and seasonal rhythmicity is available (41–43). Interestingly, both studies identified a link between chronobiological rhythmicity and pre-cluster symptoms. In the Korean and Taiwanese studies, patients who could predict their bout showed higher rates of rhythmicity compared to those who could not (71% vs. 52%, *p* = 0.019, and 72% vs. 53%, *p* = 0.009, respectively) (42,43). Taken together, these findings suggest that chronobiological rhythmicity may be associated with the timing and generation of cluster bouts, as patients who could anticipate their bouts showed clearer circadian or seasonal patterns.

## Preclinical and genetic data on the circadian features of CH

### Chronotherapeutics in CH

As mentioned in the Introduction, there are two forms of chronotherapeutics: those that are given at specific times of day and those that reset or otherwise alter the circadian clock. Circadian rhythmicity can thus be considered from a therapeutic perspective. For time of day dosing, Leone et al.(44) reported that evening administration of melatonin significantly reduced attack frequency in episodic CH, likely by stabilizing circadian rhythmicity via hypothalamic-pineal regulation. Although the efficacy of melatonin has not been validated in high-quality clinical trials, and a small study showed negative results, the findings of the study by Leone et al.(44) at least suggest the possibility that modulating circadian rhythmicity may help mitigate attack generation (45). In addition to the direct pharmacological modulation of circadian rhythmicity, aligning medication schedules with a patient’s individual circadian rhythmicity may enhance efficacy, which warrants further hypothesis-testing in future studies.

Time-of-day administration in CH, however, may eventually expand beyond giving melatonin at night. In a mouse study using the nitroglycerin headache model, ergotamine was more effective in improving mechanical hypersensitivity when given during the day than when given at night (46). One randomized controlled trial failed to demonstrate the differential efficacy of sumatriptan administered at 7:00 am, 3:00 pm and 11:00 pm (47). However, it was given on a set schedule regardless of each patient’s circadian pattern of attacks.

**Figure 5.**
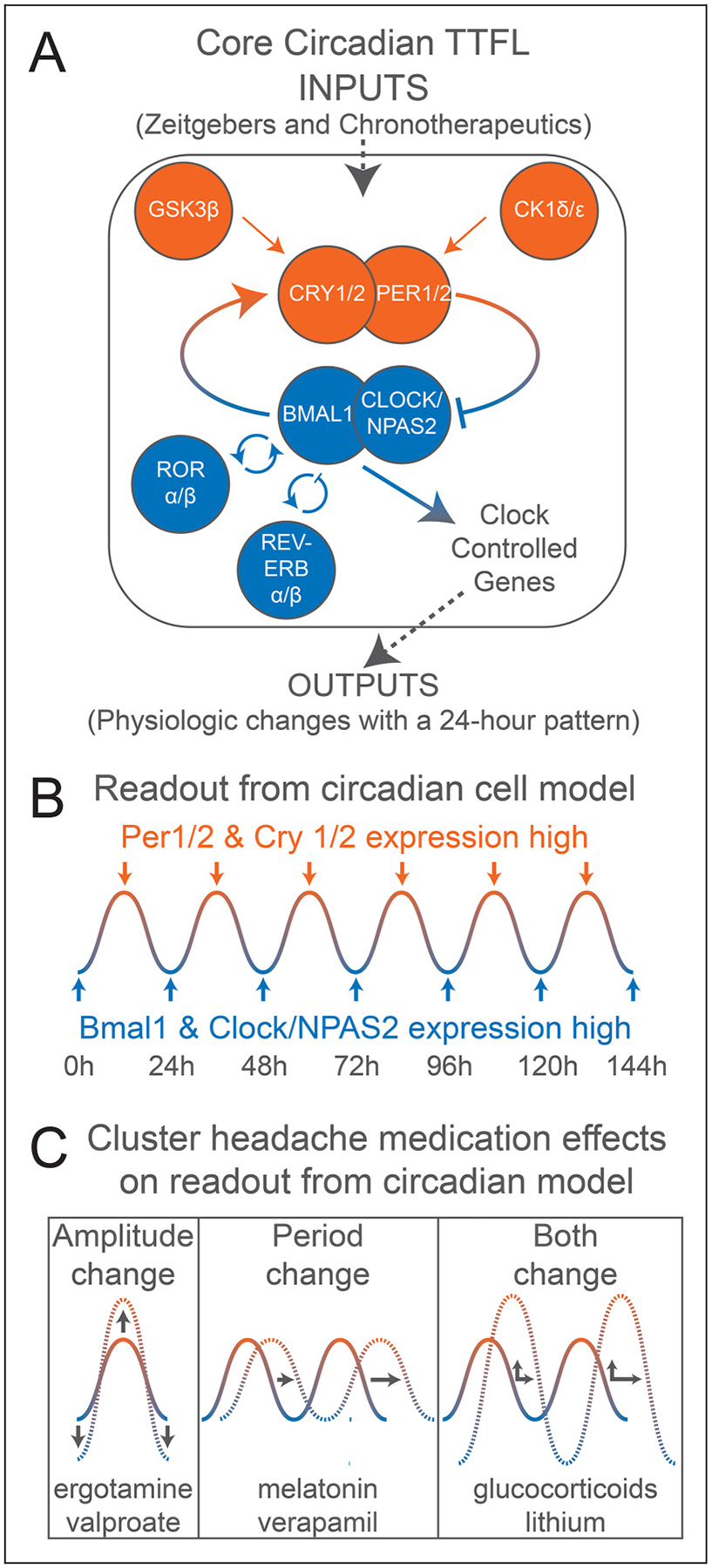
The 24-hour, intracellular, core circadian transcriptional-translational feedback loop (“TTFL”). (A) Diagrammatic representation of the TTFL. Full gene names: CLOCK = circadian locomotor output cycles kaput; NPAS2 = neuronal PAS domain protein 2, a paralog of CLOCK; BMAL1 = brain and muscle ARNT like 1; PER = period; CRY = cryptochrome; REV-ERB = reverse strand of erb; ROR = retinoid acid–related orphan receptor; CK1 = casein kinase 1; GSK3 = glycogen synthase kinase 3. (B) Sinusoidal multi-day representation of the TTFL that would be seen in circadian cell models such as Period2:: luciferase (that follows expression of Period2 over time). (C) Changes in the TTFL after various cluster headache medications are applied (references in the text). These data establish many cluster headache medications as chronotherapeutic inputs to the TTFL

The second form of chronotherapy, resetting or altering the circadian clock, has been also tested in CH. This is nowhere more evident than the use of corticosteroids (a “daytime” hormone) and melatonin (a “nighttime” hormone) as CH preventive treatments (48,49). A widely used method to study alterations in the circadian clock utilizes the Period2::luciferase reporter model (50). In this model, the core circadian gene Period2 is linked to the firefly luciferase enzyme: cells are exposed to luciferin and CH medications, placed into a luminometer, and their light emission is measured over days (Figure 5B). The amount of light emission corresponds to the amount of PER2 protein expression. Using this model, six CH preventive treatments have evidence that they alter the circadian clock (Figure 5C) (46,51–61). While these treatments have other potential mechanisms (e.g. corticosteriods are potent immunosuppressants while melatonin has sleep effects), these treatments share few other mechanisms of action and raise the idea of chronotherapy as a common mechanism for many CH preventives. Further studies are needed to understand the effects of phase and amplitude changes on downstream clock-controlled genes and on brain areas important for CH.

### Genetics and chronobiology of CH

Several genome wide association studies (GWAS) and one meta-GWAS analysis on CH have confirmed a genetic component in the aetiology of CH (62–66). None of the identified risk loci have a direct connection to circadian rhythms or to the core clock genes of the transcriptional-translational feedback loop; however, a genetic correlation between sleeplessness and insomnia was identified through linkage disequilibrium score regression (*p*-value = 4.33 × 10^−5^). The meta-GWAS comprised a total of 4777 study participants with CH and may not have power to detect smaller genetic signals and/or signals relating to a specific sub-phenotype (62). No analysis was carried out specifically on a circadian phenotype or to investigate circadian molecular pathways.

Targeted genetic analysis (i.e. candidate gene studies) have yielded several significant associations with genetic markers in genes related to the cellular and circadian clocks, or genes that were previously reported to be associated to other disorders with a circadian/circannual phenotype such as bipolar disorder, seasonal depressive disorder or sleep-related disorders. The most extensively investigated gene is the hypocretin receptor 2 (*HCRTR2*) (67). Hypocretins (or orexins) are neuropeptides synthesized in the hypothalamus and involved in circadian behaviours such as arousal, wakefulness and appetite. Several studies have investigated genetic markers, single nucleotide polymorphisms (SNPs), in the *HCRTR2* gene for associations with CH but yielded conflicting results. A recent meta-analysis did not confirm previously suggested associations with two genetic markers with reference SNP cluster ID numbers, the unique identifiers assigned to their specific locations on the human genome, rs2653349 or rs312215613, but showed a weak association with rs9357855 (68).

Other candidate gene studies have investigated genetic markers in genes central to the core circadian transcriptional-translational feedback loop (outlined in Figure 5A). Three genetic variants, rs12649507, rs11932595 and rs1801260, in the circadian locomotor output cycles kaput (*CLOCK*) gene, have been found to associate with sleep duration and chronotype in previous studies (69,70). A Swedish study identified rs12649507 as a risk variant for CH, and further found that the effect was stronger in study participants reporting that their attacks occur with a certain circadian rhythmicity (as opposed to attacks occurring randomly at any time of the day). Furthermore, the risk-allele was correlated to increased relative gene expression of the *CLOCK* gene (16). Rs1801260, which lies in the 3′ untranslated region of the *CLOCK* gene, has been repeatedly studied in relation to CH; several reports confirm that this variant is unlikely to affect the risk for CH (71–73). In addition to *CLOCK*, genetic variants in Period Circadian Regulator (*PER*) and cryptochrome (*CRY*) have been analyzed in individuals with CH. While there is no evidence for an association between CH and genetic variants in *PER* genes 1, 2 and 3 and *CRY*2, one genetic variant in *CRY1* (rs8192440) was identified as a protective variant for CH. Similar to rs12649507 in *CLOCK*, the association of rs1801260 in *CRY1* was more pronounced in study participants with a circadian phenotype of their CH attacks. In addition, increased relative *CRY1* mRNA levels were found in individuals with CH (74,75). Because the reported associations for *CLOCK* and *CRY1* were both from the same Swedish cohort, and candidate gene studies often show variable results in different populations, these associations should be replicated in other cohorts before conclusions can be drawn on the contribution of genetic variants in clock-related genes and CH. It also needs to be further investigated whether they constitute genetic risk-factors in general, or specifically for the circadian phenotype of CH as suggested by Fourier et al. (16,75). In whole genome sequencing analysis of familial CH, Popescu (76) proposed that a combination of *HCRTR2* and *CLOCK* risk-alleles confers an increased risk for CH. A notion which is also supported by haplotype analysis of *CLOCK* variants (16). A Swedish study has additionally found a genetic marker in *BMAL1*, previously associated with bipolar disorder, and a marker in *NPAS2* associated with sleep disturbances, to occur more frequently among individuals with CH (77).

### Circadian properties of CH-relevant brain areas and ganglia

Any investigation of the circadian system starts with the suprachiasmatic nucleus and the core circadian transcriptional-translational feedback loop. The suprachiasmatic nucleus is located in the anterior hypothalamus; while one structural imaging study found an enlarged anterior hypothalamus in CH patients compared to controls (78), functional imaging studies have identified the posterior nucleus of the hypothalamus, and not the suprachiasmatic nucleus, as the first area activated in a CH attack (79). The posterior hypothalamus does have connections with the suprachiasmatic nucleus, but evidence that links to the circadian system is still needed (80,81). Thus researchers have examined core circadian genes (Figure 5A). As discussed in the previous section, studies have identified core circadian gene variants in small CH populations. However, core circadian genes were not found in the larger genome wide association studies, and thus these small studies would be unlikely to explain how 70% of CH patients have a circadian pattern of attacks (10).

Because the master pacemaker (the suprachiasmatic nucleus) and core circadian genes are unlikely to explain CH’s circadian effects, the next step is to investigate other brain areas and to investigate clock-controlled genes. A meta-analysis cross-referenced three datasets: brain areas relevant for CH (a collection of brain areas identified from CH imaging review articles), tissue-specific clock-controlled genes (a baboon atlas that performed RNA sequencing on 22 brain areas and 42 non-brain tissues sampled every two hours for 24 hours) and potential CH susceptibility genes (from three GWAS). Three of the five potential CH susceptibility genes were found to cycle in five brain areas relevant for CH: prefrontal cortex/thalamus/visual cortex (*Ufl1*), cerebellum (*Mertk*) and paraventricular nucleus (*Satb2* and *Ufl1*). Subsequent to this meta-analysis being published, new CH susceptibility genes have been identified (62). Here, we used the same methodology as Benkli et al.(10) and added these new CH susceptibility genes to the imaging and baboon datasets. We found that 11 of the 18 currently identified potential CH susceptibility genes cycle in 10 brain areas relevant for CH (see Supplemental material, Table S1). The CH-relevant brain areas with the most CH cycling genes were the medial globus pallidus (the CH cycling genes were *CAPN2*, *LRP1*, *TMEM87B* and *UFL1*) and lateral globus pallidus (*KLHL32*, *LRP1*, *TMEM87B* and *UFL1*), whereas the gene that cycled in the most CH-relevant brain areas was UFL1 (in the lateral globus pallidus, medial globus pallidus, paraventricular nuclei, prefrontal cortex, thalamus and visual cortex).

Two non-brain structures, not included in the analysis above, but important in CH are the trigeminal ganglia (TG) and the upper cervical dorsal root ganglia (DRG). While not yet fully understood, both structures are clearly important in CH: sectioning of the trigeminal nerve (for the TG) and stimulation of the occipital nerve (which activates the C2 and C3 DRG), have both been reported as effective with respect to treating CH (82,83). Of note, stimulation of the sphenopalatine ganglion can also treat CH, but there is little data on the circadian properties of the sphenopalatine ganglion in pain models (84). Recently, both the TG and DRG were shown to have functional molecular clocks that are likely involved in the circadian regulation of pain (85–87). Moreover, in control mice treated with vehicle, there is no circadian pattern of hindpaw pain sensitivity (86,87). However, in the nitroglycerin animal model, which works via activation of the trigeminovascular system, mice display a 24-hour pattern of pain sensitivity that can be eliminated by abolishing the circadian system (using arrhythmic Period1/Period2 double knockout mice) (88). The nitroglycerin model, although often considered a migraine model, is arguably just as relevant as a CH model given that these disorders share mechanisms related to the trigeminovascular system (89,90). Moreover, nitroglycerin is a well-known trigger for CH attacks (91,92) and medications that work on this model, such as sumatriptan and topiramate (88,93), are effective treatments not only for migraine, but also for CH (94). In sum, the nitroglycerin model can be considered as a useful model for some of the pathophysiology (i.e. the trigeminovascular component) of CH.

Importantly, the TG clock-controlled genes undergo a large-scale transformation in response to nitroglycerin: 331 of TG genes in the control mice cease to be clock-controlled genes in the nitroglycerin-injected mice, 584 TG genes become clock-controlled genes, and only 135 TG clock-controlled genes are unchanged between the control and nitroglycerin groups (86). In the paclitaxel animal model, which induces pain via DRG fibers (95), a similar pattern is found: control mice do not have a circadian pattern of hindpaw pain sensitivity, whereas paclitaxel-injected mice do. Furthermore, DRG clock-controlled genes undergo a large-scale transformation: 696 of DRG genes in the control mice cease to be clock-controlled genes in the paclitaxel-injected mice, 1055 DRG genes become clock-controlled genes and only 136 DRG clock-controlled genes are unchanged between the control and paclitaxel groups (87).

## Conclusions and future directions

CH has circadian features in its attacks, treatments and genetics. Multiple factors including culture, sleep, chronotype, seasonal changes, temperature and inter-individual changes thus may impact CH in undiscovered ways. Future directions should focus on all of these areas, with our suggested next steps as detailed below.

### Research into circadian attacks may benefit from a clinical definition of circadian attacks that can be used for future studies

We propose to define circadian rhythmicity of CH attacks as the spontaneous onset of attacks at the same time (±1 hour) every day in which there is at least one attack, over a single bout (for episodic CH) or over three months (for chronic CH). This definition has the footnotes that: (1) the attacks meet criteria for CH; (2) during part, but less than half, of the active time-course of CH, attacks may not occur at the designated time; (3) the time of the attacks may change together with the patient’s internal clock (e.g. changing time zones) and still qualify for circadian rhythmicity; and (4) circadian rhythmicity should be subtyped as either current bout, most recent bout, lifetime or other. For research purposes, we recommend that data are preferably collected prospectively. For retrospective research, we recommend developing a structured validated question about circadian rhythmicity that takes into account that the circadian rhythmicity can change between bouts in episodic CH (to gain, lose or change the time of day) and that patients with multiple daily attacks can simultaneously have circadian and non-circadian patterns.

### Research into the circadian effects of treatments may benefit from additional preclinical testing in animal models of circadian headache such as the nitroglycerin mouse model

Human studies have started to identify the brain areas and circadian genes that may be relevant in CH. The animal model of nitroglycerin, which has relevance to CH physiologically (as a trigger of attacks) and pathophysiologically (through the trigeminovascular system), displays a 24-hour rhythm of pain sensitivity. Further exploration of this human imaging and genetic data, as well as circadian pain data in a mouse model, may be fruitful in creating a more comprehensive laboratory-based CH model.

### Research into the role of circadian genes may be better understood with larger genome wide association studies that document the presence or absence of a circadian timing of attacks

Current data linking genetics and circadian patterns of attacks are only from small studies and have found links with core circadian genes. The large-scale GWAS studies did not include data on circadian patterns of attacks. Future studies may consider circadian patterns of attacks in a subgroup analysis as there may be gene variants specific to this subgroup.

## Supplementary Material

suppl material

Supplemental material for this article is available online.

## Figures and Tables

**Table 1.** Circadian and Seasonal Rhythmicity of Cluster Headache in Previous Studies.

	Authors (Year)	Circadian	Peak time of day	Seasonal	Peak Months and Seasons	Latitude
**Systematic Review**	Benkli et al. (2023)^2^	70.5%	9pm to 3am with 2am peak (7%)	n.a.	Months: March, April, September, October and November Season: Autumn (31%)	N/A
**Country**						
United Kingdom	Bahra et al. (2002)^3^	n.a.	Nocturnal (73%) Day (37%)	n.a.	n.a.	53.8
Denmark	Barloese et al. (2015)^10,28^	82%	2am (9%)	56%	Month: November (11%)	56.2
	Lund et al. (2017)^11^	82%	n.a.	56%	n.a.	56.2
	Lund et al. (2019)^12^	50%	Nocturnal (50%)	n.a.	n.a.	56.2
Sweden	Fourier et al. (2018)^13^	66%	2am-4am	n.a.	n.a.	59.7
	Fourier et al. (2023)^14^	67%	2am-4am, Nocturnal (39%)	50%	Months: September, October and November	59.7
	Steinberg et al. (2018)^15^	67%	2am-4am (28%)	n.a.	n.a.	59.7
Netherlands	de Coo et al. (2019)^16^	72% ECH (77%) CCH (58%)	0am-4am ECH (41%), CCH (30%)	n.a.	n.a.	52.0
	van Vliet et al. (2006)^17^	64%	Nocturnal (78%)	n.a.	n.a.	52.0
Germany	Gaul et al. (2012)^18^	n.a.	1am-6am (73%)	n.a.	Seasons: Autumn (36%) and Spring (36%)	51.2
Norway	Ofte et al. (2013)^19^	58%	0am-4am (33%)	Annual (46%) Seasonal (37%)	Seasons: Spring (20%) and Autumn (13%)	61.2
	Ofte et al. (2015)^20^	n.a.	Nocturnal (51%)	n.a.	n.a.	61.2
	Russell (1981)^22^	n.a.	Nocturnal (53%) 4am-10am, peak 6am	n.a.	n.a.	61.2
Italy	Manzoni et al. (1983)^23^	n.a.	1pm-3pm	n.a.	Months: February and March Seasons: Spring and Autumn (40%)	41.6
Belgium	Verslegers et al. (2006)^24^	86%	n.a.	n.a.	n.a.	50.8
United States	Rozen & Fishman (2012)^25^	82%	2am (41%)	41%	Months: March, April, September and October, October (26%) peak Seasons: Spring and Autumn	41.3
	Urban et al. (1994)^26^	66%	10pm-4am (26%)	n.a.	Month: September (42%) Seasons: Spring and Autumn	41.3
	Kudrow (1984)^27^	n.a.	n.a.	n.a.	Months: January and July	41.3
India	Bhargava et al. (2014)^28^	83%	2pm-5pm, 12am-4am	46%	Seasons: Summer (30%) and Winter (16%)	28.7
	Chakravarty (2001)^29^	n.a.	Nocturnal (71%) Nocturnal and Day (29%)	n.a.	n.a.	28.7
South Korea	Lee et al. (2018)^30^	62%	n.a.	64%	n.a.	35.6
	Lee et al. (2020)^31^	49% (current bout) 71% (lifetime bout)	10am, 3pm and 2am	52%	n.a.	35.6
	Sohn et al. (2018)^32^	57%	Nocturnal (34%) Day (25%)	50%	Seasons: Winter to Spring (25%) and Summer to Autumn (25%)	35.6
Taiwan	Lin et al. (2004)^33^	65%	Nocturnal (28%) Day (27%)	n.a.	Months: March (13%) and December (13%),	23.7*
	Liaw et al. (2022)^34^	M: 87% F: 81%	Nocturnal (M 44%, F 52%)	M: 73% F: 71%	n.a.	23.7*
China	Dong et al. (2013)^35^	68%	7am-10am and 2pm-4pm	71%	Months: March to May (41%) and September to November (30%) Seasons: Spring (41%) and Autumn (30%)	34.5

Latitudes indicate the average latitude of a country’s northern-most and southern-most major city as reported by the United Nations (https://unstats.un.org/unsd/geoinfo/geonames/).

* Taiwanese latitudes were not available from the United Nations; as an approximation we used the average of the nearby Chinese cities Quanzhou (for the northern-most location) and Shenzhen (for the southern-most location).

Abbreviations: n.a.: Not available, M: Males, F: Females.

## Article highlights

- CH has multi-level circadian features including a clock-like pattern of attacks, variations in circadian genes in a small proportion of patients and a shared treatment mechanism (at least six CH medications alter the core circadian molecular oscillator)
- We propose several next steps to improve our understanding of the circadian features of CH: (1) a clinical definition of circadian attacks; (2) a potential animal model of circadian features of headache symptoms; and (3) study design changes to incorporate information on circadian features into genetic studies
