# Supplementary material for: Reviewing the complex relationship between circadian rhythms and cluster headache
Source: Cephalalgia. Author manuscript; Available in PMC 2026 Mar 5. (PMC12960194; doi:10.1177/03331024251365858)
Supplement: suppl material [file NIHMS2147414-supplement-suppl_material.docx]

Table S1: Map of cluster headache genes from a GWAS meta-analysis (Column A data derived from Winsvold et al. 2023. Ann. Neurol. 94(4):713) and the tissues in which they have a circadian pattern of expression in baboon tissue (Row 2 data derived from Mure et al. 2018. Science. 359(6381):eaao0318). Genes cycling in specific tissues are marked with an "x". An asterisk (*) denotes tissues relevant in the pathophysiology of cluster headache based on four imaging reviews (data analyzed in Benkli et al. 2023. Neurology 100(22):e2224).

|  | **CAPN2** | **CAPZA2** | **CFTR** | **DUSP10** | **FBLN7** | **FHL5** | **FTCDNL1** | **KLHL32** | **LRP1** | **MERTK** | **NDUFAF4** | **PLCE1** | **SATB2** | **SLC20A1** | **ST7** | **TMEM87B** | **UFL1** | **WNT2** |
| --- | --- | --- | --- | --- | --- | --- | --- | --- | --- | --- | --- | --- | --- | --- | --- | --- | --- | --- |
| **Adrenal Cortex** |  |  |  |  | x |  |  |  |  |  |  |  |  |  |  |  | x |  |
| **Adrenal Medulla** | x | x |  |  |  |  |  |  |  |  |  |  |  | x | x |  |  |  |
| **Amygdala*** |  |  |  |  |  |  |  |  |  |  |  |  |  |  |  |  |  |  |
| **Antrum** |  |  |  |  |  |  |  |  |  | x |  |  |  | x |  |  |  |  |
| **Aorta (endothelium)** |  |  |  |  |  |  |  |  |  |  |  |  | x |  | x |  | x |  |
| **Arcuate nucleus*** |  |  |  |  |  |  |  |  |  |  |  |  |  |  |  |  |  |  |
| **Ascending Colon** |  |  |  |  |  |  |  |  |  | x |  |  |  |  |  |  |  |  |
| **Axillary lymph nodes** | x |  |  |  |  |  |  |  |  |  |  |  |  |  |  |  |  |  |
| **Bladder** |  |  |  |  |  |  |  | x |  |  | x |  |  |  |  | x |  |  |
| **Bone marrow** |  |  |  |  |  |  |  |  |  |  |  |  |  |  |  |  |  |  |
| **Cecum** |  |  |  |  |  |  |  |  |  |  |  |  |  |  | x |  |  |  |
| **Cerebellum*** |  |  |  |  |  |  |  |  |  | x |  |  |  |  |  |  |  |  |
| **Cornea** |  |  |  |  |  |  |  | x |  |  |  |  |  | x |  | x |  |  |
| **Descending Colon** |  |  |  | x |  |  |  |  |  |  |  |  |  |  |  |  |  |  |
| **Dorsomedial hypothalamus*** |  |  |  |  |  |  |  |  |  |  |  |  |  |  |  |  |  |  |
| **Duodenum (opposite of ampula)** |  |  |  |  |  |  |  |  |  |  |  |  |  |  |  |  |  |  |
| **Habenula** |  |  |  |  |  |  |  |  |  |  |  |  |  |  |  |  |  |  |
| **Heart** |  | x |  |  |  |  |  |  |  | x |  |  |  |  |  |  |  |  |
| **Hippocampus*** |  |  |  |  |  |  |  |  |  |  |  |  |  |  |  |  |  |  |
| **Ileum** |  | x |  |  |  |  |  |  |  |  |  |  |  |  |  | x |  |  |
| **Iris** |  |  |  |  |  |  |  |  |  |  |  |  |  |  |  |  |  |  |
| **Kidney Cortex** |  | x |  |  |  |  |  |  |  |  |  |  |  |  | x |  |  |  |
| **Kidney Medulla** |  |  |  |  |  |  |  |  |  |  |  |  |  |  |  |  | x |  |
| **Lateral globus pallidus*** |  |  |  |  |  |  |  | x | x |  |  |  |  |  |  | x | x |  |
| **Lateral Hypothalamus*** |  |  |  |  |  |  |  |  |  |  |  |  |  |  |  |  |  |  |
| **Liver** |  |  |  |  |  |  |  |  |  |  |  |  |  |  |  |  |  |  |
| **Lungs** |  |  |  |  |  |  |  |  |  |  |  |  |  |  |  |  |  |  |
| **Medial globus pallidus*** | x |  |  |  |  |  |  |  | x |  |  |  |  |  |  | x | x |  |
| **Mesenteric Lymph nodes** |  |  |  |  |  |  |  |  |  |  |  |  |  |  |  |  |  |  |
| **Mammilary bodies** |  |  |  |  |  |  |  |  | x |  |  |  |  |  |  |  | x |  |
| **Muscle abdominal** |  |  |  |  |  |  |  |  |  |  |  | x |  | x |  |  |  |  |
| **Muscle gastrocnemian** |  |  |  |  |  |  |  |  |  |  |  |  |  |  |  |  | x |  |
| **(O)esophagus** |  |  |  |  |  |  |  |  |  |  |  |  |  | x |  |  |  |  |
| **Olfactory Bulb** |  |  |  |  |  |  |  |  |  |  |  |  |  |  |  |  |  |  |
| **Omental Fat** |  | x |  |  |  |  |  |  |  |  | x |  |  |  |  |  |  |  |
| **Optic Nerve Head** |  |  |  |  |  |  |  |  |  |  |  |  |  |  |  |  |  |  |
| **Pancreas** |  |  | x |  |  |  |  |  |  | x |  |  |  |  |  | x |  |  |
| **Paraventricular Nuclei*** |  | x |  |  |  |  |  |  |  |  |  |  | x |  |  |  | x |  |
| **Pineal** |  |  |  |  |  |  |  |  |  |  |  |  |  |  |  |  |  |  |
| **Pituitary** |  |  |  |  |  |  |  |  |  |  |  |  |  |  |  |  |  |  |
| **Pons*** |  |  |  |  |  |  |  |  | x |  |  |  |  |  |  | x |  |  |
| **Preoptic Area*** |  |  |  |  |  |  |  |  |  |  |  |  |  |  |  |  |  |  |
| **Prefrontal cortex*** |  |  |  |  |  |  |  |  | x |  | x |  |  |  |  |  | x |  |
| **Prostate** |  |  |  |  |  |  |  |  |  |  |  |  |  |  |  |  |  |  |
| **Putamen*** |  |  |  |  |  |  |  |  |  |  |  |  |  |  | x |  |  |  |
| **Retina** |  |  |  |  |  |  |  |  |  | x |  |  |  |  |  |  |  |  |
| **Retinal Pigment Epithelium** |  |  |  |  |  |  |  |  |  |  |  |  |  |  |  |  | x |  |
| **Suprachiasmatic Nuclei*** |  |  |  |  |  |  |  |  |  |  |  |  |  |  |  |  |  |  |
| **Skin from underbelly** |  |  |  |  |  |  |  |  | x |  |  |  |  |  |  |  |  |  |
| **Smooth muscle (opposite to antrum)** |  |  |  |  |  | x |  |  |  |  |  |  |  |  |  |  |  |  |
| **Supraoptic nucleus*** |  |  |  |  |  |  |  |  |  |  |  |  |  |  |  |  |  |  |
| **Spleen** |  |  |  |  |  |  |  |  |  |  |  |  |  |  |  |  |  |  |
| **Stomach Fundus** |  | x |  |  |  |  |  |  |  |  | x |  |  |  |  |  | x |  |
| **Substantia nigra*** |  |  |  |  |  |  |  |  |  |  |  |  |  |  |  |  |  |  |
| **Testicles** |  |  |  |  |  |  |  |  |  |  |  |  |  |  |  | x | x |  |
| **Thalamus*** |  |  |  |  |  |  |  |  |  |  |  |  |  |  |  |  | x |  |
| **Thyroid** |  | x |  |  |  |  |  | x | x |  | x |  |  |  |  |  |  |  |
| **Visual cortex*** |  | x |  |  |  |  |  |  |  |  |  |  |  |  |  |  | x |  |
| **Ventromedial hypothalamus*** |  |  |  |  |  |  |  |  | x |  |  |  |  | x |  |  |  |  |
| **White adipose mesenteric** |  |  |  |  |  |  |  |  |  |  |  |  |  |  |  |  |  |  |
| **White adipose pericardial** |  | x |  |  |  |  |  |  |  |  |  |  |  |  |  |  | x |  |
| **White adipose perirenal** |  |  |  |  |  |  |  |  |  |  |  |  |  |  |  |  |  |  |
| **White adipose subcutaneous** |  |  |  |  |  |  |  |  |  |  |  |  |  |  |  |  |  |  |
| **White adipose retroperitoneal** |  |  |  |  |  |  |  |  |  |  |  |  |  |  |  |  |  |  |
